# Supplementary material for: Cyclophosphamide addition to pomalidomide/dexamethasone is not necessarily associated with universal benefits in RRMM
Source: PLoS One. 2022 Jan 27;17(1):e0260113. doi: 10.1371/journal.pone.0260113 (PMC8794080; doi:10.1371/journal.pone.0260113)
Supplement: S1 Table — (DOCX) [file pone.0260113.s001.docx]

**S1 Table.** Progression free survival and overall survival according to the subgroups

|  | | 2-year PFS (%) | | | | 2-year OS (%) | | | |
| --- | --- | --- | --- | --- | --- | --- | --- | --- | --- |
| Variables | | Pd | | PCd | P value | Pd | | PCd | P value |
| Age, years | >68 | | 41.4 (±14.2) | 32.4 (±11.3) | 0.920 | 47.1 (±12.6) | | 54.7 (±10.3) | 0.665 |
|  | ≤68 | | 26.0 (±8.2) | 40.8 (±14.5) | 0.165 | 61.0 (±9.6) | | 34.8 (±13.4) | 0.176 |
| ECOG | 0, 1 | | 28.0 (±7.7) | 30.5 (±9.5) | 0.159 | 57.3 (±8.2) | | 48.6 (±8.7) | 0.189 |
|  | >2 | | 45.7 (±22.4) | 37.5 (±28.6) | 0.814 | 45.7 (±22.4) | | 66.7 (±27.2) | 0.255 |
| Extramedullary disease | Presence | | 55.6 (±16.6) | 51.9 (±17.7) | 0.455 | 100 | | 53.0 (±15.5) | 0.150 |
|  | Absence | | 24.0 (±9.8) | 20.3 (±8.0) | 0.155 | 44.8 (±8.9) | | 44.6 (±10.1) | 0.893 |
| R-ISS stage | 1 | | 60.0 (±21.9) | 66.7 (±27.2) | 0.796 | 80.0 (±17.9) | | 30.0 (±23.9) | 0.214 |
|  | 2 | | 43.3 (±13.4) | 28.6 (±15.8) | 0.665 | 55.6 (±11.3) | | 37.6 (±12.7) | 0.663 |
|  | 3 | | 0 | 0 | 0.665 | | 38.9 (±19.2) | 0 | 0.382 |
| High risk myeloma [18] | High-risk | | 31.9 (±9.1) | 39.7 (±11.9) | 0.229 | | 53.7 (±9.1) | 42.7 (±10.7) | 0.738 |
|  | None | | 27.0 (±12.2) | 21.0 (±12.5) | 0.225 | | 61.0 (±14.2) | 59.9 (±13.4) | 0.309 |
| Cytogenetics | High | | 17.7 (±11.3) | 0 | 0.179 | | 29.8 (±15.2) | 0 | 0.432 |
|  | Standard | | 30.3 (±10.8) | 32.3 (±12.1) | 0.128 | | 76.2 (±9.3) | 44.0 (±11.0) | 0.139 |
| Time from diagnosis to | >49months | | 34.1 (±9.6) | 41.6 (±15.6) | 0.295 | | 58.0 (±10.1) | 67.7 (±14.0) | 0.488 |
| Pomalidomide | ≤49months | | 19.2 (±11.3) | 29.7 (±10.3) | 0.293 | | 53.8 (±11.7) | 35.8 (±10.0) | 0.146 |
| Previous treatment lines | ≥4 | | 23.3 (±9.1) | 22.3 (±13.6) | 0.137 | | 56.4 (±11.4) | 51.1 (±16.4) | 0.979 |
|  | <4 | | 35.8 (±11.9) | 40.6 (±10.6) | 0.680 | | 55.5 (±10.4) | 44.3 (±9.9) | 0.240 |
| Previous autoSCT | Done | | 30.0 (±9.1) | 23.6 (±14.5) | 0.342 | | 61.4 (±10.5) | 41.3 (±16.6) | 0.333 |
|  | Not done | | 25.6 (±13.3) | 39.5 (±10.2) | 0.327 | | 49.2 (±11.2) | 48.8 (±9.8) | 0.965 |
| Previous thalidomide | CR/VGPR | | 25.0 (±21.7) | 60.0 (±21.9) | 0.055 | | 100 | 68.6 (±18.6) | 0.143 |
| response | PR-PD | | 0 | 37.5 (±19.8) | 0.121 | | 55.4 (±15.4) | 42.2 (±15.7) | 0.291 |
| Previous lenalidomide | CR/VGPR | | 25.0 (20.4) | 0 | 0.531 | | 57.1 (±18.7) | 40.0 (±21.9) | 0.674 |
| response | PR-PD | | 31.0 (8.1) | 35.8 (±9.6) | 0.109 | | 55.0 (±8.6) | 51.3 (±9.0) | 0.522 |
| Lenalidomide PFS | ≥26months | | 52.5 (±17.6) | 75.0 (±21.7) | 0.854 | | 65.6 (±16.4) | 66.7 (±27.2) | 0.892 |
| (Upper 15% of PFS) | < 26months | | 21.8 (±7.8) | 28.4 (±9.9) | 0.048 | | 55.4 (±8.7) | 46.8 (±9.6) | 0.292 |
| Previous bortezomib | CR/VGPR | | 26.0 (±14.3) | 37.0 (±15.4) | 0.409 | | 58.4 (±14.6) | 41.1 (±12.7) | 0.145 |
| response | PR-PD | | 31.8 (±9.2) | 28.0 (±10.8) | 0.454 | | 56.8 (±9.5) | 49.8 (±11.5) | 0.963 |

Abbreviations: PFS=progression free survival; OS=overall survival; Pd= pomalidomide+dexamethasone; PCd= pomalidomide+cyclophophsamide+dexamethasone; ECOG=Eastern Cooperative Oncology Group performance status; R-ISS=Revised International Staging System; autoSCT=autologous stem cell transplantation; CR=complete response; VGPR= very good partial response; PR=partial response; PD=progressive disease.
