# Supplementary material for: Cyclophosphamide addition to pomalidomide/dexamethasone is not necessarily associated with universal benefits in RRMM
Source: PLoS One. 2022 Jan 27;17(1):e0260113. doi: 10.1371/journal.pone.0260113 (PMC8794080; doi:10.1371/journal.pone.0260113)
Supplement: S2 Table — (DOCX) [file pone.0260113.s002.docx]

**S2 Table.** Baseline characteristics, response and survival outcomes of patients in Pd→PCd group

| **Patient characteristics** | Pd→PCd (17 patients) |
| --- | --- |
| Age (median, range), years | 67 (51–85) |
| Previous exposure to bortezomib, n (%) | 17 (100) |
| Previous exposure to lenalidomide, n (%) | 17 (100) |
| Extramedullary disease, n (%)  Presence  Absence | 3 (17.6)  14 (82.4) |
| R-ISS stage, n (%)  1  2  3  Unknown | 3 (17.6)  7 (41.2)  1 (5.9)  6 (35.3) |
| Cytogenetic high risk, n (%) | 1/10 (10.0) |
| Months from diagnosis to pomalidomide, median (range) | 52 (9–134) |
| Previous lines of therapy, median (range) | 4 (2–5) |
| Previous autoSCT, n (%) | 7 (41.1) |
| Median cycles of treatment, median (range)  **Total (Pd+PCd)**  Pd  PCd | **14 (4–30)**  6 (1–17)  6 (2–24) |
| **Response** |  |
| Overall response rate during Pd + PCd, n (%)  VGPR  PR  SD | 3 (17.6)  9 (52.9)  5 (29.4) |
| Overall response rate during Pd, n (%) |  |
| VGPR | 2 (11.8) |
| PR | 5 (29.4) |
| SD | 10 (58.8) |
| Overall response rate during PCd, n (%)  VGPR  PR  SD | 3 (17.6)  7 (41.2)  7 (41.2) |
| Change in overall response from Pd to PCd, n (%)  **Improved response**  SD🡪VGPR  SD🡪 PR  **No change in response**  VGPR🡪VGPR  PR🡪PR  SD🡪SD  **Poor response**  PR🡪SD | **5 (29.4)**  1 (5.9)  4 (23.5)  **10 (58.8)**  2 (11.8)  3 (17.6)  5 (29.4)  **2 (11.8)**  2 (11.8) |
| **Survival** |  |
| PFS, months, median (95% confidence interval) |  |
| PFS of Pd | 4.0 (1.6**–**6.4) |
| PFS of PCd | 10.0 (4.4-15.6) |
| OS, months, median (95% confidence interval) |  |
| OS of Pd  OS of PCd | 28.0 (23.5-32.5)  14.0 (6.6-21.4) |

Abbreviations: Pd= pomalidomide+dexamethasone; PCd= pomalidomide+cyclophophsamide+dexamethasone; R-ISS= Revised International Staging System; autoSCT= autologous stem cell transplantation; VGPR= very good partial response; PR= partial response; SD= stable disease; PFS= progression free survival; OS= overall survival.
