# Supplementary material for: Cyclophosphamide addition to pomalidomide/dexamethasone is not necessarily associated with universal benefits in RRMM
Source: PLoS One. 2022 Jan 27;17(1):e0260113. doi: 10.1371/journal.pone.0260113 (PMC8794080; doi:10.1371/journal.pone.0260113)
Supplement: S3 Table — (DOCX) [file pone.0260113.s003.docx]

**S3 Table.** The overall response rates (ORR) and predictive factors for ORR (Intention-to-treatment analysis)

| **Variables (n, %)** |  | **Pd**  **(*N*=74)** | **PCd**  **(*N*=29)** | ***p*** |
| --- | --- | --- | --- | --- |
| **Response rates** | ORR | 32/65 (49.2) | 22/28 (78.6) | 0.009 |
|  | sCR or CR | 1/65 (1.5) | 2/28 (7.1) | 0.215 |
|  | VGPR | 5/65 (7.7) | 1/28 (3.6) | 0.664 |
|  | PR | 26/65 (40.0) | 19/28 (67.9) | 0.014 |
|  | SD | 30/65 (46.2) | 6/28 (21.4) | 0.025 |
|  | PD | 3/65 (4.6) | 0 | 0.551 |
| **Age, years** | >68 | 14/25 (56.0) | 14/18 (77.8) | 0.199 |
|  | ≤68 | 18/40 (45.0) | 8/10 (80.0) | 0.077 |
| **ECOG** | 0, 1 | 26/55 (47.3) | 20/26 (76.9) | 0.012 |
|  | >2 | 6/10 (60.0) | 2/2 (100) | 0.515 |
| **Extramedullary disease** | Presence | 4/11 (36.4) | 7/7 (100) | 0.013 |
|  | Absence | 28/54 (51.9) | 15/21 (71.4) | 0.124 |
| **R-ISS stage** | 1 | 3/7 (42.9) | 2/2 (100) | 0.444 |
|  | 2 | 18/29 (62.1) | 8/12 (66.7) | 1.000 |
|  | 3 | 3/10 (30.0) | 5/6 (83.3) | 0.119 |
| **High risk myeloma [18]** | High-risk | 17/41 (41.5) | 14/17 (82.4) | 0.008 |
|  | None | 15/24 (62.5) | 8/11 (72.7) | 0.709 |
| **Cytogenetics** | High | 6/14 (42.9) | 6/6 (100) | 0.042 |
|  | Standard | 13/30 (43.3) | 12/16 (75.0) | 0.063 |
| **Time from diagnosis to pom** | >49 months | 22/39 (56.4) | 4/5 (80.0) | 0.634 |
|  | ≤49 months | 10/26 (38.5) | 18/23 (78.3) | 0.009 |
| **Previous treatment lines** | ≥4 | 16/32 (50.0) | 3/5 (60.0) | 1.000 |
|  | <4 | 16/33 (48.5) | 19/23 (82.6) | 0.012 |
| **Previous autoSCT** | Done | 16/34 (47.1) | 4/7 (57.1) | 0.697 |
|  | Not done | 16/31 (51.6) | 18/21 (85.7) | 0.017 |
| **Previous thalidomide response** | CR/VGPR | 2/5 (40.0) | 4/4 (100) | 0.167 |
|  | PR-PD | 9/17 (52.9) | 6/8 (75.0) | 0.402 |
| **Previous lenalidomide response** | CR/VGPR | 4/9 (44.4) | 2/3 (66.7) | 1.000 |
|  | PR-PD | 27/55 (49.1) | 20/25 (80.0) | 0.009 |
| **Lenalidomide PFS*** | ≥26months | 6/9 (66.7) | 1/2 (50.0) | 1.000 |
|  | <26months | 25/55 (45.5) | 18/21 (85.7) | 0.002 |
| **Previous bortezomib response** | CR/VGPR | 12/21 (57.1) | 9/12 (75.0) | 0.457 |
|  | PR-PD | 18/41 (43.9) | 13/16 (81.3) | 0.017 |

*Cut-off of 26 months was used because this was the upper 15% lenalidomide PFS.

Abbreviations:Pd=pomalidomide+dexamethasone/pomalidomide+dexamethasone🡪pomalidomide+cyclophosphamide+dexamethasone; PCd= pomalidomide+cyclophosphamide+dexamethasone; ORR= overall response rate; sCR= stringent CR; CR= complete response; VGPR= very good partial response; PR= partial response; SD= stable disease; PD= progressive disease; ECOG= Eastern Cooperative Oncology Group performance status; R-ISS= Revised International Staging System; Pom= pomalidomide; autoSCT= autologous stem cell transplantation; PFS= progression free survival.
