# Supplementary material for: Cyclophosphamide addition to pomalidomide/dexamethasone is not necessarily associated with universal benefits in RRMM
Source: PLoS One. 2022 Jan 27;17(1):e0260113. doi: 10.1371/journal.pone.0260113 (PMC8794080; doi:10.1371/journal.pone.0260113)
Supplement: S4 Table — (DOCX) [file pone.0260113.s004.docx]

**S4 Table**. Progression free survival and overall survival in all patients (Intention-to-treatment analysis)

| **Variables** | | **Univariate** | | **Multivariate** | | **Univariate** | | **Multivariate** | |
| --- | --- | --- | --- | --- | --- | --- | --- | --- | --- |
|  |  | Median PFS  (95% CI) | *p* | HR (95% CI) | *p* | Median OS  (95% CI) | *p* | HR (95% CI) | *p* |
| **Age, years** | >68 | 14.5 (10.5–18.4) | 0.537 |  |  | 25.0 (18.4–31.5) | 0.282 |  |  |
|  | ≤68 | 12.3 (6.7–17.9) |  |  |  | 25.0 (11.5–38.5) |  |  |  |
| **ECOG** | 0, 1 | 14.0 (10.8–17.2) | 0.657 |  |  | 25.0 (17.2–32.8) | 0.815 |  |  |
|  | >2 | 10.6 (9.8–11.4) |  |  |  | 28.6 (8.5–48.8) |  |  |  |
| **Extramedullary disease** | Presence | 13.3 (9.2–17.3) | 0.276 |  |  | 19.8 (13.3–26.2) | 0.048 | 1.904 (0.749–4.836) | 0.176 |
|  | Absence | 27.1 (0.1–54.3) |  |  |  | 47.3 (–) |  | 1 |  |
| **R-ISS stage** | 1 | 21.0 (0.1–55.3) | <0.001 | 1 |  | 25.0 (1.3–48.6) | 0.222 |  |  |
|  | 2 | 18.2 (11.1–25.3) |  | 2.191 (0.746–6.433) | 0.153 | 23.1 (15.5–30.8) |  |  |  |
|  | 3 | 6.1 (3.6–8.7) |  | 6.777(1.966–23.357) | 0.002 | 13.0 (4.7–21.3) |  |  |  |
| **High risk myeloma[18]** | High-risk | 13.5 (8.5–18.4) | 0.961 |  |  | 19.8 (11.4–28.1) | 0.320 |  |  |
|  | None | 14.0 (9.0–19.0) |  |  |  | 25.3 (20.7–29.8) |  |  |  |
| **Cytogenetics** | Poor | 9.3 (3.6–14.9) | 0.103 |  |  | 13.3 (3.4–23.2) | 0.014 | 2.572 (1.171–5.646) | 0.019 |
|  | Standard | 13.5 (7.8–19.2) |  |  |  | 25.0 (20.1–29.8) |  | 1 |  |
| **Cyclophophamide** | Added | 14.0 (11.7–16.3) | 0.932 |  |  | 14.9 (6.0–23.7) | 0.040 | 2.446 (1.151–5.200) | 0.020 |
|  | Not added | 13.3 (7.8–18.7) |  |  |  | 27.8 (23.2–32.4) |  | 1 |  |
| **Dx to pomalidomide** | >49months | 14.0 (3.3–24.7) | 0.481 |  |  | 27.8 (22.9–32.8) | 0.313 |  |  |
|  | ≤49months | 13.3 (9.2–17.3) |  |  |  | 18.8 (10.9–26.7) |  |  |  |
| **Previous treatment lines** | ≥4 | 14.0 (7.6–20.4) | 0.517 |  |  | 25.3 (17.0–33.5) | 0.717 |  |  |
|  | <4 | 13.5 (7.0–20.0) |  |  |  | 23.1 (11.4–45.0) |  |  |  |
| **Previous autoSCT** | Done | 13.3 (7.0–19.5) | 0.621 |  |  | 25.3 (16.7–33.8) | 0.268 |  |  |
|  | Not done | 14.5 (9.9–19.0) |  |  |  | 23.1 (12.5–33.8) |  |  |  |
| **Previous thalidomide response** | CR/VGPR | 16.6 (0.1–40.6) | 0.075 |  |  | 47.3 (–) | 0.087 |  |  |
|  | PR-PD | 13.3 (9.8–16.7) |  |  |  | 18.8 (0.1–38.5) |  |  |  |
| **Previous lenalidomide response** | CR/VGPR | 16.6 (4.1–29.1) | 0.920 |  |  | 17.7 (4.4–31.1) | 0.882 |  |  |
|  | PR-PD | 13.5 (10.2–16.7) |  |  |  | 25.0 (18.5–31.4) |  |  |  |
| **Previous bortezomib response** | CR/VGPR | 14.5 (9.7–19.2) | 0.410 |  |  | 23.6 (12.6–34.5) | 0.581 |  |  |
|  | PR-PD | 13.3 (6.8–19.7) |  |  |  | 27.5 (16.1–38.9) |  |  |  |
| **Pomalidomide response** | sCR-PR | 18.2 (8.2–28.2) | <0.001 | 1 |  | 23.1 (14.3–32.0) | 0.033 | 1 | 0.043 |
|  | SD/PD | 5.5 (1.3–9.8) |  | 5.540 (2.600–11.804) | <0.001 | Not reached |  | 2.354 (1.027–5.395) |  |

Abbreviations: PFS= progression free survival; OS= overall survival; HR= hazard ratio; CI= confidence interval; ECOG= Eastern Cooperative Oncology Group performance status; R-ISS= Revised International Staging System; Dx= diagnosis; autoSCT= autologous stem cell transplantation; sCR= stringent complete response; CR= complete response; VGPR= very good partial response; PR= partial response; SD= stable disease; PD= progressive disease.
