# Supplementary material for: Cyclophosphamide addition to pomalidomide/dexamethasone is not necessarily associated with universal benefits in RRMM
Source: PLoS One. 2022 Jan 27;17(1):e0260113. doi: 10.1371/journal.pone.0260113 (PMC8794080; doi:10.1371/journal.pone.0260113)
Supplement: S1 Fig — (DOCX) [file pone.0260113.s005.docx]

**S1 Fig.** CONSORT flow diagram.

Abbreviations: Pom=pomalidomide; dexa=dexamethasone
