# Supplementary material for: Cyclophosphamide addition to pomalidomide/dexamethasone is not necessarily associated with universal benefits in RRMM
Source: PLoS One. 2022 Jan 27;17(1):e0260113. doi: 10.1371/journal.pone.0260113 (PMC8794080; doi:10.1371/journal.pone.0260113)

**S2 Fig.** (A) Progression-free survival (PFS) and (B) overall survival (OS) according to the treatment response to pomalidomide-based therapy (2-year PFS: 45.8±9.0% for sCR–PR vs. 13.3±6.0% for SD and PD, P<0.001; 2-year OS: 47.9±7.4% for sCR–PR vs. 68.1±9.3% for SD and PD, P=0.005).

Abbreviations: sCR=stringent complete response=sCR; CR=complete response; VGPR= very good partial response; PR=partial response; SD=stable disease; PD=progressive disease.


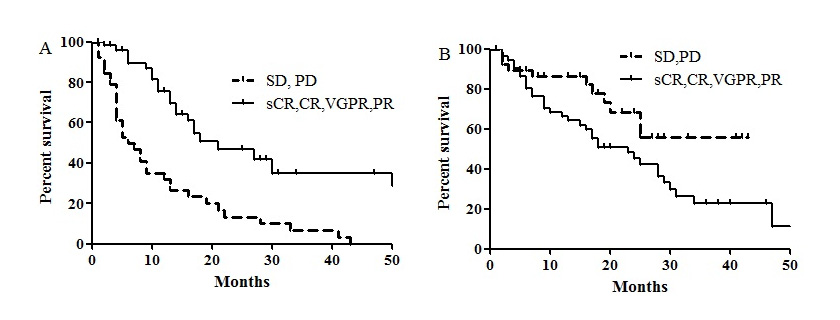

Supplement: S2 Fig — (DOCX) [file pone.0260113.s006.docx]
