# Supplementary material for: Cyclophosphamide addition to pomalidomide/dexamethasone is not necessarily associated with universal benefits in RRMM
Source: PLoS One. 2022 Jan 27;17(1):e0260113. doi: 10.1371/journal.pone.0260113 (PMC8794080; doi:10.1371/journal.pone.0260113)

**S3 Fig.** Progression-free survival after pomalidomide-based chemotherapy (median PFS: 159 days for carfilzomib-based vs. 29 days for daratumumab-based vs. 28 days for bendamustine-based. 186 days for DCEP vs. 34 days for other therapy, P=0.022).

Abbreviations: DCEP = dexamethasone + cyclophosphamide + etoposide + cisplatin; Other therapy = melphalan-based for 2 patients, thalidomide-based for 2 patients, bortezomib-based for 1 patient, cyclophosphamide-based chemotherapy for 1 patient.


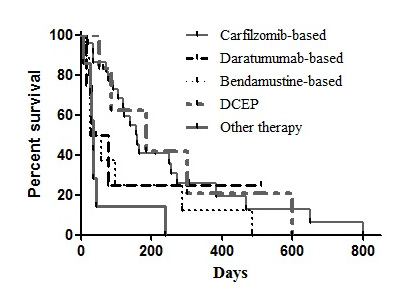

Supplement: S3 Fig — (DOCX) [file pone.0260113.s007.docx]
