# Supplementary material for: Cyclophosphamide addition to pomalidomide/dexamethasone is not necessarily associated with universal benefits in RRMM
Source: PLoS One. 2022 Jan 27;17(1):e0260113. doi: 10.1371/journal.pone.0260113 (PMC8794080; doi:10.1371/journal.pone.0260113)
Supplement: Attachment — Submitted filename: Review Comments to the Author_pomalidomide_final version.docx [file pone.0260113.s008.docx]

**Dear Editors**

We wish to express our gratitude to you and the reviewers for the careful review of our manuscript entitled **‘Cyclophosphamide addition to pomalidomide/dexamethasone is not necessarily associated with universal benefits in RRMM.’** The thoughtful comments provided valuable advices to improve the manuscript. We have revised our manuscript based on the reviewers’ comments. Here, we have addressed the comments of the reviewers, as well as our responses to the specific comments. The revised portions of the main manuscript are recorded with red colored text in the revised manuscript. We hope that you and the reviewers will find this revision satisfactory. We look forward to having our manuscript published in ***PLOS ONE.***

Thank you again for the timely and constructive review of our manuscript.

Best regards,

**Correspondence autors**

Sung-Soo Yoon, M.D., Ph.D.

Department of Internal Medicine, Seoul National University Hospital, 101 Daehak-ro, Jongno-gu, Seoul 03080, Korea

E-mail: ssysmc@snu.ac.kr; Tel: +82-2-2072-3079; Fax: +82-2-762-9662

Youngil Koh, MD, PhD

Department of Internal Medicine, Seoul National University Hospital, 101 Daehak-ro, Jongno-gu, Seoul 03080, Korea

E-mail: go01@snu.ac.kr; Tel: 82-2-2072-7217, Fax: 82-2-762-9662

**Review Comments to the Author

Please use the space provided to explain your answers to the questions above. You may also include additional comments for the author, including concerns about dual publication, research ethics, or publication ethics. (Please upload your review as an attachment if it exceeds 20,000 characters)**

**Reviewer #1: I read with interest the manuscript by Park et al. The authors studied in retrospective study the added value of adding CTX to pomalidomide and dexamethasone for relapsed refractory MM patients. The authors concluded that adding CTX increases response rate but no survival outcome. to note, that majority of response enhancement is at a level of partial response. The authors also found that patients with high-risk cytogenetics and poorer response to prior IMiD increases response to added CTX.
The paper is overall well written and easy to follow.
I have several concerns as outlined below:**

**1. The Two groups (Pd, PCd) are not balanced with regard to age, number of prior lines of therapy/months from diagnosis to Pomalidomide initiation and prior ASCT. This is a major caveat and the authors should try and explain these differences and highlight it in the text. This is one of the unavoidable limitation of retrospective study, and number of patients (n=103) is too small to partially overcomes these biases.**

🡪 We thank the reviewer for their helpful comments. As you note, patient characteristics differed between Pd and PCd groups, which is a limitation of our retrospective study and allows for potential bias that could influence the results. Therefore, future studies that employ more rigorous designs with larger numbers of patients are needed to confirm our results. To address this point, we added the following sentence to the revised manuscript:

**<Page 12 Line 219–222>**

(Added) First, our study included a small number of patients and had uneven distributions of characteristics between groups, allowing the possibility that bias could influence our results. Thus, studies employing more rigorous designs with larger numbers of patients are needed to confirm our results.

**2. The authors performed MVA to find independent predictors to PFS and OS. However, the used 2-year PFS/OS which is less informative. The authors should have perform time-to-event analysis. Also choosing P-value of 0.05 in the univariate analysis is very restrictive. I am not sure the authors have enough power for MVA, but being under-powered does not justify less than satisfactory analysis.**

🡪 Thank you for your comment. We previously performed time-to-event analysis using Kaplan-Meier method mentioned, as noted in page 6 Line 79. However, as you noted, As the results of 2-year PFS/OS may not be highly informative, we re-analyzed median PFS/OS (Table 3). We also re-performed multivariate analysis using clinical variables with *p-*values <0.1 in univariate analyses. However, the results of this re-analysis may be less informative due to many clinical variables relative to the small sample size in each subgroup. Therefore, we only used variables with *p-*values <0.05 in the final multivariate analysis.

**<Table 3: Final version>**

**Table 3**. Progression free survival and overall survival in all patients

| **Variables** | | **Univariate** | | **Multivariate** | | **Univariate** | | **Multivariate** | |
| --- | --- | --- | --- | --- | --- | --- | --- | --- | --- |
|  |  | Median PFS  (95% CI) | *p* | HR (95% CI) | *p* | Median OS  (95% CI) | *p* | HR (95% CI) | *p* |
| **Age, years** | >68 | 14.5 (10.5–18.4) | 0.537 |  |  | 25.0 (18.4–31.5) | 0.282 |  |  |
|  | ≤68 | 12.3 (6.7–17.9) |  |  |  | 25.0 (11.5–38.5) |  |  |  |
| **ECOG** | 0, 1 | 14.0 (10.8–17.2) | 0.657 |  |  | 25.0 (17.2–32.8) | 0.815 |  |  |
|  | >2 | 10.6 (9.8–11.4) |  |  |  | 28.6 (8.5–48.8) |  |  |  |
| **Extramedullary disease** | Presence | 13.3 (9.2–17.3) | 0.276 |  |  | 19.8 (13.3–26.2) | 0.048 | 1.628 (0.645–4.112) | 0.302 |
|  | Absence | 27.1 (0.1–54.3) |  |  |  | 47.3 (–) |  | 1 |  |
| **R-ISS stage** | 1 | 21.0 (0.1–55.3) | <0.001 | 1 |  | 25.0 (1.3–48.6) | 0.222 |  |  |
|  | 2 | 18.2 (11.1–25.3) |  | 2.191 (0.746–6.433) | 0.153 | 23.1 (15.5–30.8) |  |  |  |
|  | 3 | 6.1 (3.6–8.7) |  | 6.777(1.966–23.357) | 0.002 | 13.0 (4.7–21.3) |  |  |  |
| **High risk myeloma[18]** | High-risk | 13.5 (8.5–18.4) | 0.961 |  |  | 19.8 (11.4–28.1) | 0.320 |  |  |
|  | None | 14.0 (9.0–19.0) |  |  |  | 25.3 (20.7–29.8) |  |  |  |
| **Cytogenetics** | Poor | 9.3 (3.6–14.9) | 0.103 |  |  | 13.3 (3.4–23.2) | 0.014 | 2.158 (1.005–4.633) | 0.048 |
|  | Standard | 13.5 (7.8–19.2) |  |  |  | 25.0 (20.1–29.8) |  | 1 |  |
| **Cyclophophamide** | Added | 16.6 (14.8–18.4) | 0.162 |  |  | 23.6 (14.7–32.5) | 0.358 |  |  |
|  | Not added | 9.8 (6.1–13.5) |  |  |  | 28.6 (13.9–43.4) |  |  |  |
| **Dx to pomalidomide** | >49months | 14.0 (3.3–24.7) | 0.481 |  |  | 27.8 (22.9–32.8) | 0.313 |  |  |
|  | ≤49months | 13.3 (9.2–17.3) |  |  |  | 18.8 (10.9–26.7) |  |  |  |
| **Previous treatment lines** | ≥4 | 14.0 (7.6–20.4) | 0.517 |  |  | 25.3 (17.0–33.5) | 0.717 |  |  |
|  | <4 | 13.5 (7.0–20.0) |  |  |  | 23.1 (11.4–45.0) |  |  |  |
| **Previous autoSCT** | Done | 13.3 (7.0–19.5) | 0.621 |  |  | 25.3 (16.7–33.8) | 0.268 |  |  |
|  | Not done | 14.5 (9.9–19.0) |  |  |  | 23.1 (12.5–33.8) |  |  |  |
| **Previous thalidomide response** | CR/VGPR | 16.6 (0.1–40.6) | 0.075 |  |  | 47.3 (–) | 0.087 |  |  |
|  | PR-PD | 13.3 (9.8–16.7) |  |  |  | 18.8 (0.1–38.5) |  |  |  |
| **Previous lenalidomide response** | CR/VGPR | 16.6 (4.1–29.1) | 0.920 |  |  | 17.7 (4.4–31.1) | 0.882 |  |  |
|  | PR-PD | 13.5 (10.2–16.7) |  |  |  | 25.0 (18.5–31.4) |  |  |  |
| **Previous bortezomib response** | CR/VGPR | 14.5 (9.7–19.2) | 0.410 |  |  | 23.6 (12.6–34.5) | 0.581 |  |  |
|  | PR-PD | 13.3 (6.8–19.7) |  |  |  | 27.5 (16.1–38.9) |  |  |  |
| **Pomalidomide response** | sCR-PR | 18.2 (8.2–28.2) | <0.001 | 1 |  | 23.1 (14.3–32.0) | 0.033 | 1 | 0.008 |
|  | SD/PD | 5.5 (1.3–9.8) |  | 5.540 (2.600–11.804) | <0.001 | Not reached |  | 2.938 (1.325–6.518) |  |

Abbreviations: PFS= progression free survival; OS= overall survival; HR= hazard ratio; CI= confidence interval; ECOG= Eastern Cooperative Oncology Group performance status; R-ISS= Revised International Staging System; Dx= diagnosis; autoSCT= autologous stem cell transplantation; sCR= stringent complete response; CR= complete response; VGPR= very good partial response; PR= partial response; SD= stable disease; PD= progressive disease.

**<Table 3: Review only: clinical variables with *p-*values <0.1 >**

**Table 3**. Progression free survival and overall survival in all patients (Review only)

| **Variables** | | **Univariate** | | **Multivariate** | | **Univariate** | | **Multivariate** | |
| --- | --- | --- | --- | --- | --- | --- | --- | --- | --- |
|  |  | Median PFS  (95% CI) | *p* | HR (95% CI) | *p* | Median OS  (95% CI) | *p* | HR (95% CI) | *p* |
| **Age, years** | >68 | 14.5 (10.5–18.4) | 0.537 |  |  | 25.0 (18.4–31.5) | 0.282 |  |  |
|  | ≤68 | 12.3 (6.7–17.9) |  |  |  | 25.0 (11.5–38.5) |  |  |  |
| **ECOG** | 0, 1 | 14.0 (10.8–17.2) | 0.657 |  |  | 25.0 (17.2–32.8) | 0.815 |  |  |
|  | >2 | 10.6 (9.8–11.4) |  |  |  | 28.6 (8.5–48.8) |  |  |  |
| **Extramedullary disease** | Presence | 13.3 (9.2–17.3) | 0.276 |  |  | 19.8 (13.3–26.2) | 0.048 | 1.377 (0.211–9.000) | 0.738 |
|  | Absence | 27.1 (0.1–54.3) |  |  |  | 47.3 (–) |  | 1 |  |
| **R-ISS stage** | 1 | 21.0 (0.1–55.3) | <0.001 | 1 |  | 25.0 (1.3–48.6) | 0.222 |  |  |
|  | 2 | 18.2 (11.1–25.3) |  | 0.681 (0.063–7.295) | 0.750 | 23.1 (15.5–30.8) |  |  |  |
|  | 3 | 6.1 (3.6–8.7) |  | 2.115 (0.118–38.066) | 0.611 | 13.0 (4.7–21.3) |  |  |  |
| **High risk myeloma[18]** | High-risk | 13.5 (8.5–18.4) | 0.961 |  |  | 19.8 (11.4–28.1) | 0.320 |  |  |
|  | None | 14.0 (9.0–19.0) |  |  |  | 25.3 (20.7–29.8) |  |  |  |
| **Cytogenetics** | Poor | 9.3 (3.6–14.9) | 0.103 |  |  | 13.3 (3.4–23.2) | 0.014 | 1.264 (0.270–5.911) | 0.766 |
|  | Standard | 13.5 (7.8–19.2) |  |  |  | 25.0 (20.1–29.8) |  | 1 |  |
| **Cyclophophamide** | Added | 16.6 (14.8–18.4) | 0.162 |  |  | 23.6 (14.7–32.5) | 0.358 |  |  |
|  | Not added | 9.8 (6.1–13.5) |  |  |  | 28.6 (13.9–43.4) |  |  |  |
| **Dx to pomalidomide** | >49months | 14.0 (3.3–24.7) | 0.481 |  |  | 27.8 (22.9–32.8) | 0.313 |  |  |
|  | ≤49months | 13.3 (9.2–17.3) |  |  |  | 18.8 (10.9–26.7) |  |  |  |
| **Previous treatment lines** | ≥4 | 14.0 (7.6–20.4) | 0.517 |  |  | 25.3 (17.0–33.5) | 0.717 |  |  |
|  | <4 | 13.5 (7.0–20.0) |  |  |  | 23.1 (11.4–45.0) |  |  |  |
| **Previous autoSCT** | Done | 13.3 (7.0–19.5) | 0.621 |  |  | 25.3 (16.7–33.8) | 0.268 |  |  |
|  | Not done | 14.5 (9.9–19.0) |  |  |  | 23.1 (12.5–33.8) |  |  |  |
| **Previous thalidomide response** | CR/VGPR | 16.6 (0.1–40.6) | 0.075 | 1 | 0.112 | 47.3 (–) | 0.087 | 1 | 0.636 |
|  | PR-PD | 13.3 (9.8–16.7) |  | 9.112 (0.598–138.824) |  | 18.8 (0.1–38.5) |  | 1.545 (0.255–9.377) |  |
| **Previous lenalidomide response** | CR/VGPR | 16.6 (4.1–29.1) | 0.920 |  |  | 17.7 (4.4–31.1) | 0.882 |  |  |
|  | PR-PD | 13.5 (10.2–16.7) |  |  |  | 25.0 (18.5–31.4) |  |  |  |
| **Previous bortezomib response** | CR/VGPR | 14.5 (9.7–19.2) | 0.410 |  |  | 23.6 (12.6–34.5) | 0.581 |  |  |
|  | PR-PD | 13.3 (6.8–19.7) |  |  |  | 27.5 (16.1–38.9) |  |  |  |
| **Pomalidomide response** | sCR-PR | 18.2 (8.2–28.2) | <0.001 | 1 |  | 23.1 (14.3–32.0) | 0.033 | 1 | 0.199 |
|  | SD/PD | 5.5 (1.3–9.8) |  | 19.420 (1.649–228.758) | 0.018 | Not reached |  | 2.991 (0.562–15.919) |  |

Abbreviations: PFS= progression free survival; OS= overall survival; HR= hazard ratio; CI= confidence interval; ECOG= Eastern Cooperative Oncology Group performance status; R-ISS= Revised International Staging System; Dx= diagnosis; autoSCT= autologous stem cell transplantation; sCR= stringent complete response; CR= complete response; VGPR= very good partial response; PR= partial response; SD= stable disease; PD= progressive disease.

**3. 17 patients (out of 46 patients in the PCd group) started with Pd and CTX was added at a later stage. This is a major bias, since we do not know what CTX would add to the comparative group. The authors should list in detail the results of this subgroup, including their baseline characteristics, response (survival is indeed listed).**

🡪 As you note, 17 patients received additional cyclophosphamide after a median of six cycles of Pd. We added the baseline characteristics of this Pd→PCd group to S2 Table. The best response of these 17 patients during treatment with Pd regimen was as follows: VGPR=2/17, PR=5/17, SD=10/17. All patients were given additional cyclophosphamide due to increased M protein before progressive disease. Of these patients, 10 did not achieve a better response than the best response during Pd treatment (S2 Table; VGPR→VGPR for 2 patients, PR→PR for 3 patients, and SD→SD for 5 patients); 5 showed an improved response (SD→VGPR for 1 patient and SD→PR for 4 patients); and 2 showed a poor response (PR→SD). However, rather than improving response, we found that the addition of cyclophosphamide may prolong PFS in heavily treated patients. This interpretation must be considered with caution due to our small sample size and definition of PFS for the Pd regimen as the time from administration of Pd to the addition of cyclophosphamide, rather than definite progression. To clarify these issues, we added text to the revised manuscript as follows:

**<Page 6 Line 68–69>**

(Added) PFS for the Pd regimen in Pd→PCd group was defined as the time from administration of Pd to the addition of cyclophosphamide.

**<Page 8 Line 129–Page 9 Line 136>**

(Added) *Response and survival outcomes in Pd→PCd group*

We further analyzed patient characteristics, response, and survival in Pd→PCd group (17 patients). All patients in this group received additional cyclophosphamide due to increased M protein before progressive disease. ORR was unchanged in most patients (58.8%) after the addition of cyclophosphamide (VGPR→VGPR for 2/17 patients, PR→PR for 3/17 patients, and SD→SD for 5/17 patients; S2 Table). However, PFS was significantly prolonged after the addition of cyclophosphamide compared with the Pd-only regimen (median 4.0 months for Pd vs. 10.0 months for PCd; S2 Table).

**<Page 11 Line 197–Page 12 Line 202>**

(Added) One important aspect of our study is that some patients treated with Pd received additional cyclophosphamide (Pd→PCd group). In these patients, the addition of cyclophosphamide before progressive disease tended to prolong the treatment period but did not achieve a significantly improved response (S2 Table). However, we found that cyclophosphamide may delay disease progression in patients whose disease is gradually worsening.

**(Added) S2 Table.** Baseline characteristics, response and survival outcomes of patients in Pd→PCd group

| **Patient characteristics** | Pd→PCd (17 patients) |
| --- | --- |
| Age (median, range), years | 67 (51–85) |
| Previous exposure to bortezomib, n (%) | 17 (100) |
| Previous exposure to lenalidomide, n (%) | 17 (100) |
| Extramedullary disease, n (%)  Presence  Absence | 3 (17.6)  14 (82.4) |
| R-ISS stage, n (%)  1  2  3  Unknown | 3 (17.6)  7 (41.2)  1 (5.9)  6 (35.3) |
| Cytogenetic high risk, n (%) | 1/10 (10.0) |
| Months from diagnosis to pomalidomide, median (range) | 52 (9–134) |
| Previous lines of therapy, median (range) | 4 (2–5) |
| Previous autoSCT, n (%) | 7 (41.1) |
| Median cycles of treatment, median (range)  **Total (Pd+PCd)**  Pd  PCd | **14 (4–30)**  6 (1–17)  6 (2–24) |
| **Response** |  |
| Overall response rate during Pd + PCd, n (%)  VGPR  PR  SD | 3 (17.6)  9 (52.9)  5 (29.4) |
| Overall response rate during Pd, n (%) |  |
| VGPR | 2 (11.8) |
| PR | 5 (29.4) |
| SD | 10 (58.8) |
| Overall response rate during PCd, n (%)  VGPR  PR  SD | 3 (17.6)  7 (41.2)  7 (41.2) |
| Change in overall response from Pd to PCd, n (%)  **Improved response**  SD🡪VGPR  SD🡪 PR  **No change in response**  VGPR🡪VGPR  PR🡪PR  SD🡪SD  **Poor response**  PR🡪SD | **5 (29.4)**  1 (5.9)  4 (23.5)  **10 (58.8)**  2 (11.8)  3 (17.6)  5 (29.4)  **2 (11.8)**  2 (11.8) |
| **Survival** |  |
| PFS, months, median (95% confidence interval) |  |
| PFS of Pd | 4.0 (1.6**–**6.4) |
| PFS of PCd | 10.0 (4.4-15.6) |
| OS, months, median (95% confidence interval) |  |
| OS of Pd  OS of PCd | 28.0 (23.5-32.5)  14.0 (6.6-21.4) |

Abbreviations: Pd= pomalidomide+dexamethasone; PCd= pomalidomide+cyclophophsamide+dexamethasone; R-ISS= Revised International Staging System; autoSCT= autologous stem cell transplantation; VGPR= very good partial response; PR= partial response; SD= stable disease; PFS= progression free survival; OS= overall survival.

**Reviewer #2: Review of article D-21-18676
This represents a retrospective review that attempts to compare patients treated with doublet pomalidomide dexamethasone with those treated with triplet cyclophosphamide pomalidomide dexamethasone.
The authors attempt to compare 57 in the doublet group compared with 46 in the triplet group and conclude that response rate was higher in the triplet group and try to identify subsets that benefit with the addition of cyclophosphamide.**

**Please clarify why patients receiving the triplet received an oral serotonin antagonist. This is found on page 5**
🡪 We thank the reviewer for their helpful comments. In the 2021 NCCN guidelines, cyclophosphamide <1500 mg/m^2^ is considered to have moderate emetic risk. Therefore, we added an oral serotonin antagonist to the PCd regimen. To address this point, we revised the text and added a reference as follows:

**< Page 5 Line 54–56>**

(Original manuscript) Patients in PCd group received additional oral serotonin antagonist on days 1, 8, and 15.

(Revised manuscript) Patients in PCd group also received an oral serotonin antagonist on days 1, 8, and 15 due to the moderate emetic risk associated with cyclophosphamide [15].

<References>

15. Network NCC. Antiemesis. 2021.

**The authors will need to reconsider the analysis because the groups they are reporting are not “intent to treat”. Nearly 35% of the patients in the pomalidomide cyclophosphamide dexamethasone group had 17 patients where cyclophosphamide was added after median of 6 cycles of pomalidomide dexamethasone. This distorts the statistical analysis. This was clearly “intent to treat” with a doublet and the addition of cyclophosphamide suggest these patients had an inadequate response to the doublet so this represents clear reporting bias. Moreover, patients would need to survive for 6 months in order to begin cyclophosphamide which will impact their report of progression-free and overall survival making it look better for triplet group since the timing of progression-free survival would have been initiation of the doublet 6 cycles earlier. The report of an overall response rate of 75.6% in the triplet group would also be biased based on what I assume would be a failure to respond to the doublet and the addition of pomalidomide at 6 months.

Therefore, the analysis really needs to be intention to treat and the 17 patients that had cyclophosphamide added after 6 months belong to the doublet group of pomalidomide dexamethasone. I suspect this will have a profound impact on the interpretation of the data. Since the median pomalidomide progression-free survival was 13.5 months and pomalidomide was added to 17 patients after 6 months this will almost certainly decline significantly.**

🡪 As noted by the reviewer, 17 patients received additional cyclophosphamide after a median of six cycles of pomalidomide + dexamethasone. All of these patients received additional cyclophosphamide due to increased M protein before progressive disease. Therefore, this may have biased the PCd group toward better PFS, OS, and ORR. As recommended by the reviewer, we re-classified patients in the Pd→PCd group based on their initial treatment regimen and re-analyzed the data (S3 Tables 3 and 4). In this intention-to treatment analysis, ORR was superior in the PCd group (78.6%) compared with the Pd group (49.2%, *p=*0.009), but PFS was similar between groups (median: 13.3 months for Pd vs. 14.0 months for PCd, *p=*0.932). OS was prolonged in the Pd group compared with the PCd group (median: 27.8 months for Pd vs. 14.9 months for PCd, *p=*0.040). Because PFS was similar between the Pd and PCd groups, we suspected that subsequent treatment might prolong OS. To describe this analysis, we added new text to the revised manuscript as follows:

**<Page 6 Line 71–72>**

(Added) Intention-to treatment analysis was performed by grouping patients based on their initial treatment regimens.

**<Page 9 Line 138–147>**

(Added) *Intention-to-treatment analysis*

We performed intention-to-treatment analysis according to patients’ initial treatment regimens (Pd group: Pd or Pd→PCd regimens vs. PCd group). PCd group showed significantly better ORR than Pd group (78.6% vs. 49.2%. respectively, *p*=0.009; S3 Table). Subgroup analysis showed that patients with better ECOG performance status (0 or 1), extramedullary disease, high-risk myeloma, previous treatment lines < 4, or poor response to lenalidomide and bortezomib benefitted from additional cyclophosphamide (S3 Table). In survival analysis, Pd group showed better OS than PCd group, but PFS was similar between groups (median OS: 27.8 months for Pd group vs. 14.9 months for PCd group, *p*= 0.040; median PFS: 13.3 months for Pd group vs. 14.0 months for PCd group, *p*=0.932; S4 Table).

**(Added) S3 Table.** The overall response rates (ORR) and predictive factors for ORR

| **Variables (n, %)** |  | **Pd**  **(*N*=74)** | **PCd**  **(*N*=29)** | ***p*** |
| --- | --- | --- | --- | --- |
| **Response rates** | ORR | 32/65 (49.2) | 22/28 (78.6) | 0.009 |
|  | sCR or CR | 1/65 (1.5) | 2/28 (7.1) | 0.215 |
|  | VGPR | 5/65 (7.7) | 1/28 (3.6) | 0.664 |
|  | PR | 26/65 (40.0) | 19/28 (67.9) | 0.014 |
|  | SD | 30/65 (46.2) | 6/28 (21.4) | 0.025 |
|  | PD | 3/65 (4.6) | 0 | 0.551 |
| **Age, years** | >68 | 14/25 (56.0) | 14/18 (77.8) | 0.199 |
|  | ≤68 | 18/40 (45.0) | 8/10 (80.0) | 0.077 |
| **ECOG** | 0, 1 | 26/55 (47.3) | 20/26 (76.9) | 0.012 |
|  | >2 | 6/10 (60.0) | 2/2 (100) | 0.515 |
| **Extramedullary disease** | Presence | 4/11 (36.4) | 7/7 (100) | 0.013 |
|  | Absence | 28/54 (51.9) | 15/21 (71.4) | 0.124 |
| **R-ISS stage** | 1 | 3/7 (42.9) | 2/2 (100) | 0.444 |
|  | 2 | 18/29 (62.1) | 8/12 (66.7) | 1.000 |
|  | 3 | 3/10 (30.0) | 5/6 (83.3) | 0.119 |
| **High risk myeloma [18]** | High-risk | 17/41 (41.5) | 14/17 (82.4) | 0.008 |
|  | None | 15/24 (62.5) | 8/11 (72.7) | 0.709 |
| **Cytogenetics** | High | 6/14 (42.9) | 6/6 (100) | 0.042 |
|  | Standard | 13/30 (43.3) | 12/16 (75.0) | 0.063 |
| **Time from diagnosis to pom** | >49 months | 22/39 (56.4) | 4/5 (80.0) | 0.634 |
|  | ≤49 months | 10/26 (38.5) | 18/23 (78.3) | 0.009 |
| **Previous treatment lines** | ≥4 | 16/32 (50.0) | 3/5 (60.0) | 1.000 |
|  | <4 | 16/33 (48.5) | 19/23 (82.6) | 0.012 |
| **Previous autoSCT** | Done | 16/34 (47.1) | 4/7 (57.1) | 0.697 |
|  | Not done | 16/31 (51.6) | 18/21 (85.7) | 0.017 |
| **Previous thalidomide response** | CR/VGPR | 2/5 (40.0) | 4/4 (100) | 0.167 |
|  | PR-PD | 9/17 (52.9) | 6/8 (75.0) | 0.402 |
| **Previous lenalidomide response** | CR/VGPR | 4/9 (44.4) | 2/3 (66.7) | 1.000 |
|  | PR-PD | 27/55 (49.1) | 20/25 (80.0) | 0.009 |
| **Lenalidomide PFS*** | ≥26months | 6/9 (66.7) | 1/2 (50.0) | 1.000 |
|  | <26months | 25/55 (45.5) | 18/21 (85.7) | 0.002 |
| **Previous bortezomib response** | CR/VGPR | 12/21 (57.1) | 9/12 (75.0) | 0.457 |
|  | PR-PD | 18/41 (43.9) | 13/16 (81.3) | 0.017 |

(Intention-to-treatment analysis)

*Cut-off of 26 months was used because this was the upper 15% lenalidomide PFS.

**(Added) S4 Table**. Progression free survival and overall survival in all patients (Intention-to-treatment analysis)

| **Variables** | | **Univariate** | | **Multivariate** | | **Univariate** | | **Multivariate** | |
| --- | --- | --- | --- | --- | --- | --- | --- | --- | --- |
|  |  | Median PFS  (95% CI) | *p* | HR (95% CI) | *p* | Median OS  (95% CI) | *p* | HR (95% CI) | *p* |
| **Age, years** | >68 | 14.5 (10.5–18.4) | 0.537 |  |  | 25.0 (18.4–31.5) | 0.282 |  |  |
|  | ≤68 | 12.3 (6.7–17.9) |  |  |  | 25.0 (11.5–38.5) |  |  |  |
| **ECOG** | 0, 1 | 14.0 (10.8–17.2) | 0.657 |  |  | 25.0 (17.2–32.8) | 0.815 |  |  |
|  | >2 | 10.6 (9.8–11.4) |  |  |  | 28.6 (8.5–48.8) |  |  |  |
| **Extramedullary disease** | Presence | 13.3 (9.2–17.3) | 0.276 |  |  | 19.8 (13.3–26.2) | 0.048 | 1.904 (0.749–4.836) | 0.176 |
|  | Absence | 27.1 (0.1–54.3) |  |  |  | 47.3 (–) |  | 1 |  |
| **R-ISS stage** | 1 | 21.0 (0.1–55.3) | <0.001 | 1 |  | 25.0 (1.3–48.6) | 0.222 |  |  |
|  | 2 | 18.2 (11.1–25.3) |  | 2.191 (0.746–6.433) | 0.153 | 23.1 (15.5–30.8) |  |  |  |
|  | 3 | 6.1 (3.6–8.7) |  | 6.777(1.966–23.357) | 0.002 | 13.0 (4.7–21.3) |  |  |  |
| **High risk myeloma[18]** | High-risk | 13.5 (8.5–18.4) | 0.961 |  |  | 19.8 (11.4–28.1) | 0.320 |  |  |
|  | None | 14.0 (9.0–19.0) |  |  |  | 25.3 (20.7–29.8) |  |  |  |
| **Cytogenetics** | Poor | 9.3 (3.6–14.9) | 0.103 |  |  | 13.3 (3.4–23.2) | 0.014 | 2.572 (1.171–5.646) | 0.019 |
|  | Standard | 13.5 (7.8–19.2) |  |  |  | 25.0 (20.1–29.8) |  | 1 |  |
| **Cyclophophamide** | Added | 14.0 (11.7–16.3) | 0.932 |  |  | 14.9 (6.0–23.7) | 0.040 | 2.446 (1.151–5.200) | 0.020 |
|  | Not added | 13.3 (7.8–18.7) |  |  |  | 27.8 (23.2–32.4) |  | 1 |  |
| **Dx to pomalidomide** | >49months | 14.0 (3.3–24.7) | 0.481 |  |  | 27.8 (22.9–32.8) | 0.313 |  |  |
|  | ≤49months | 13.3 (9.2–17.3) |  |  |  | 18.8 (10.9–26.7) |  |  |  |
| **Previous treatment lines** | ≥4 | 14.0 (7.6–20.4) | 0.517 |  |  | 25.3 (17.0–33.5) | 0.717 |  |  |
|  | <4 | 13.5 (7.0–20.0) |  |  |  | 23.1 (11.4–45.0) |  |  |  |
| **Previous autoSCT** | Done | 13.3 (7.0–19.5) | 0.621 |  |  | 25.3 (16.7–33.8) | 0.268 |  |  |
|  | Not done | 14.5 (9.9–19.0) |  |  |  | 23.1 (12.5–33.8) |  |  |  |
| **Previous thalidomide response** | CR/VGPR | 16.6 (0.1–40.6) | 0.075 |  |  | 47.3 (–) | 0.087 |  |  |
|  | PR-PD | 13.3 (9.8–16.7) |  |  |  | 18.8 (0.1–38.5) |  |  |  |
| **Previous lenalidomide response** | CR/VGPR | 16.6 (4.1–29.1) | 0.920 |  |  | 17.7 (4.4–31.1) | 0.882 |  |  |
|  | PR-PD | 13.5 (10.2–16.7) |  |  |  | 25.0 (18.5–31.4) |  |  |  |
| **Previous bortezomib response** | CR/VGPR | 14.5 (9.7–19.2) | 0.410 |  |  | 23.6 (12.6–34.5) | 0.581 |  |  |
|  | PR-PD | 13.3 (6.8–19.7) |  |  |  | 27.5 (16.1–38.9) |  |  |  |
| **Pomalidomide response** | sCR-PR | 18.2 (8.2–28.2) | <0.001 | 1 |  | 23.1 (14.3–32.0) | 0.033 | 1 | 0.043 |
|  | SD/PD | 5.5 (1.3–9.8) |  | 5.540 (2.600–11.804) | <0.001 | Not reached |  | 2.354 (1.027–5.395) |  |

**Finally, retrospective trials are notoriously poor in reporting toxicity since it requires documentation by the provider on side effects that may not have been pre specified in the trial** **such as nausea vomiting diarrhea weight loss etc the discussion should indicate that in a retrospective trial documenting toxicity is not particularly reliable.**

🡪 We agree that reports of toxicity may be underestimated in retrospective studies. To address this point, we added text to the revised manuscript as follows:

**<Page 12 Line 224–Page 13 Line 225>**

(Added) Third, evaluation of adverse events was limited because only documented reports could be analyzed.

Additionally, we previously evaluated nausea/vomiting, but did not included in Table 5. Therefore, we added to the Table 5 as follows:

**(Revised) Table 5**. Adverse events

| **Adverse events, n (%)** | **All patients**  **(*N*=103)** | **Pd**  **(*N*=57)** | **PCd**  **(*N*=46)** | ***p*** |
| --- | --- | --- | --- | --- |
| Neutropenia (≥gr 3) | 47/103 (56.7) | 25/57 (43.9) | 22/46 (47.8) | 0.613 |
| Anemia (≥gr 3) | 25/103 (24.3) | 14/57 (24.6) | 11/46 (23.9) | 0.989 |
| Thrombocytopenia (≥gr 3) | 31/103 (30.1) | 20/57 (35.1) | 11/46 (23.9) | 0.219 |
| Neutropenic fever | 24/103 (23.3) | 11/57 (19.3) | 13/46 (28.3) | 0.285 |
| Pneumonia | 48/103 (46.6) | 23/57 (40.4) | 25/46 (54.3) | 0.157 |
| Sepsis | 7/103 (6.8) | 3/57 (5.3) | 4/46 (8.7) | 0.697 |
| Kidney injury | 8/103 (7.8) | 6/57 (10.5) | 2/46 (4.3) | 0.293 |
| PPN (≥gr 3) | 1/103 (1.0) | 1/57 (1.8) | 0/46 (0) | 1.000 |
| Peripheral edema (≥gr 3) | 1/103 (1.0) | 1/57 (1.8) | 0/46 (0) | 1.000 |
| Nausea/Vomiting  Constipation | 9/103 (8.7)  15/103 (14.6) | 6/57 (10.5)  9/57 (15.8) | 3/46 (6.5)  6/46 (13.0) | 0.728  0.694 |
| Diarrhea | 10/103 (9.7) | 6/57 (10.5) | 4/46 (8.7) | 1.000 |
